# Supplementary material for: Refinement of the “Candidatus Accumulibacter” genus based on metagenomic analysis of biological nutrient removal (BNR) pilot-scale plants operated with reduced aeration
Source: mSystems. 2024 Feb 28;9(3):e01188-23. doi: 10.1128/msystems.01188-23 (PMC10949500; doi:10.1128/msystems.01188-23)
Supplement: Supplemental Figures and Tables — Figures S1, S2, and S3 and Tables S1 and S2. [file msystems.01188-23-s0001.docx]

***Supplementary document for:***

**Refinement of the “*Candidatus* Accumulibacter” Genus Based on a Metagenomic Analysis of Biological Nutrient Removal (BNR) Pilot-Scale Plants Operated with Reduced Aeration**

Rachel D. Stewart^a^, Kevin S. Myers^b,c^, Carly Amstadt^a^, Matt Seib^d^, Katherine D. McMahon^a,b,e^, Daniel R. Noguera^a,b,c*^

^a^Department of Civil and Environmental Engineering, University of Wisconsin-Madison, Madison, WI, USA

^b^Wisconsin Energy Institute, University of Wisconsin-Madison, Madison, WI, USA

^c^Great Lakes Bioenergy Research Center, University of Wisconsin-Madison, Madison, WI, USA

^d^Madison Metropolitan Sewerage District, Madison, WI, USA

^e^Department of Bacteriology, University of Wisconsin-Madison, Madison, WI, USA

^*^Corresponding author: noguera@engr.wisc.edu

**Figure S1.** Clusters of the UW14 to UW29 MAGs along with the reference genomes for the “*Ca*. Accumulibacter” species, generated by clustering analysis using dRep and a 95% ANI genome-wide threshold for cluster definition. Cluster designations are denoted inside the parentheses.


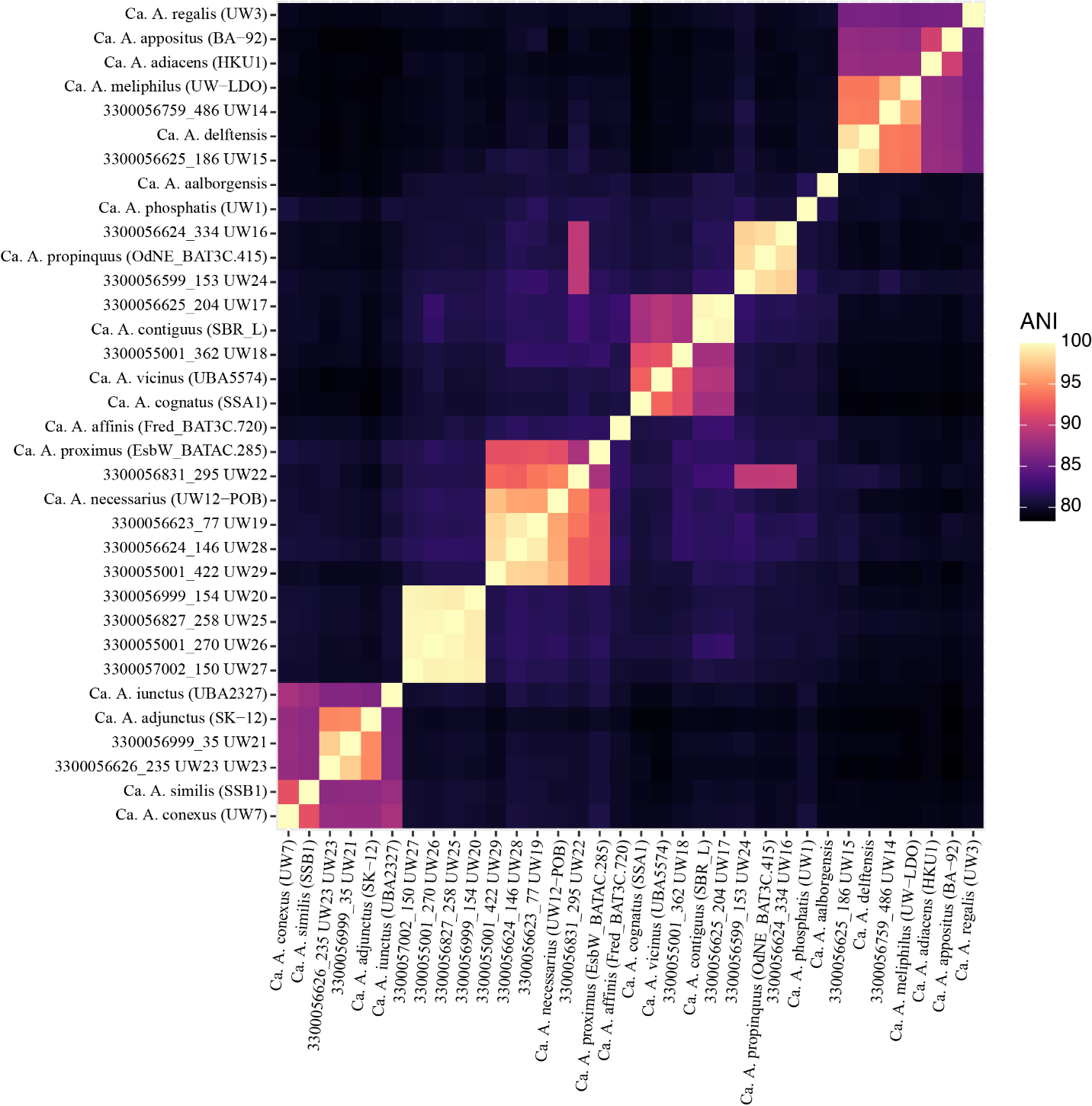


**Figure S2**. Pairwise genome-wide ANI comparisons made with fastANI (1) between the Accumulibacter MAGs assembled in this study (UW14 to UW29) and the reference genomes of the current “*Ca.* Accumulibacter” species (2).

**Figure S3.** Phylogenetic tree of a fragment of the non-redundant *ppk1* gene sequence from a database compiled by McDaniel et al. (3), supplemented with sequences from Petriglieri et al. (2) and the UW14 to UW29 Accumulibacter MAGs assembled in this study. The tree was constructed RAxML-NG using 100 bootstraps. Bolded tips represent *ppk1* sequences recovered from MAGs assembled in this study.

**Table S1.** Metagenome Summary and Identification (ID) Numbers for the Metagenomes and the Assemblies

| **Pilot Plant** | **Sample Collection Date** | **Day of Operation** | **Sample_Name** | **Project ID** | **Metagenome Analysis ID** | **IMG taxon ID** | **NCBI SRA Biosample Accession** | **Sequencing Platform** |
| --- | --- | --- | --- | --- | --- | --- | --- | --- |
| AOia | 2019-10-02 | 155 | RS_2019-10-02_SampleA | [Gp0648936](https://gold.jgi.doe.gov/project?id=Gp0648936) | [Ga0558115](https://gold.jgi.doe.gov/analysis_project?id=Ga0558115) | [3300056926](https://img.jgi.doe.gov/cgi-bin/mer/main.cgi?section=TaxonDetail&page=taxonDetail&taxon_oid=3300056926) | [SAMN37864930](https://www.ncbi.nlm.nih.gov/sra/?term=SAMN37864930) | PacBio 3kb |
| AOia | 2019-10-09 | 162 | RS_2019-10-09_SampleA | [Gp0615585](https://gold.jgi.doe.gov/project?id=Gp0615585) | [Ga0558720](https://gold.jgi.doe.gov/analysis_project?id=Ga0558720) | [3300056789](https://img.jgi.doe.gov/cgi-bin/mer/main.cgi?section=TaxonDetail&page=taxonDetail&taxon_oid=3300056789) | [SAMN37864924](https://www.ncbi.nlm.nih.gov/sra/?term=SAMN37864924) | PacBio 6-10kb |
| AOia | 2019-10-23 | 176 | RS_2019-10-23_SampleA | [Gp0615586](https://gold.jgi.doe.gov/project?id=Gp0615586) | [Ga0530630](https://gold.jgi.doe.gov/analysis_project?id=Ga0530630) | [3300053043](https://img.jgi.doe.gov/cgi-bin/mer/main.cgi?section=TaxonDetail&page=taxonDetail&taxon_oid=3300053043) | [SAMN37864931](https://www.ncbi.nlm.nih.gov/sra/?term=SAMN37864931) | PacBio 6-10kb |
| AOia | 2020-02-05 | 281 | RS_2020-02-05_SampleA | [Gp0615587](https://gold.jgi.doe.gov/project?id=Gp0615587) | [Ga0569291](https://gold.jgi.doe.gov/analysis_project?id=Ga0569291) | [3300056855](https://img.jgi.doe.gov/cgi-bin/mer/main.cgi?section=TaxonDetail&page=taxonDetail&taxon_oid=3300056855) | [SAMN37864942](https://www.ncbi.nlm.nih.gov/sra/?term=SAMN37864942) | PacBio 3kb |
| AOia | 2020-02-12 | 288 | RS_2020-02-12_SampleA | [Gp0648937](https://gold.jgi.doe.gov/project?id=Gp0648937) | [Ga0558116](https://gold.jgi.doe.gov/analysis_project?id=Ga0558116) | [3300056623](https://img.jgi.doe.gov/cgi-bin/mer/main.cgi?section=TaxonDetail&page=taxonDetail&taxon_oid=3300056623) | [SAMN37864934](https://www.ncbi.nlm.nih.gov/sra/?term=SAMN37864934) | PacBio 6-10kb |
| AOia | 2020-02-26 | 302 | RS_2020-02-26_SampleA | [Gp0615588](https://gold.jgi.doe.gov/project?id=Gp0615588) | [Ga0563660](https://gold.jgi.doe.gov/analysis_project?id=Ga0563660) | [3300057002](https://img.jgi.doe.gov/cgi-bin/mer/main.cgi?section=TaxonDetail&page=taxonDetail&taxon_oid=3300057002) | [SAMN37864940](https://www.ncbi.nlm.nih.gov/sra/?term=SAMN37864940) | Illumina |
| AOia | 2020-07-02 | 429 | RS_2020-07-02_SampleA | [Gp0648939](https://gold.jgi.doe.gov/project?id=Gp0648939) | [Ga0558118](https://gold.jgi.doe.gov/analysis_project?id=Ga0558118) | [3300056827](https://img.jgi.doe.gov/cgi-bin/mer/main.cgi?section=TaxonDetail&page=taxonDetail&taxon_oid=3300056827) | [SAMN37864939](https://www.ncbi.nlm.nih.gov/sra/?term=SAMN37864939) | PacBio 6-10kb |
| AOia | 2020-07-14 | 441 | RS_2020-07-14_SampleA | [Gp0615589](https://gold.jgi.doe.gov/project?id=Gp0615589) | [Ga0556162](https://gold.jgi.doe.gov/analysis_project?id=Ga0556162) | [3300055001](https://img.jgi.doe.gov/cgi-bin/mer/main.cgi?section=TaxonDetail&page=taxonDetail&taxon_oid=3300055001) | [SAMN37864922](https://www.ncbi.nlm.nih.gov/sra/?term=SAMN37864922) | Illumina |
| AOia | 2021-01-20 | 631 | RS_2021-01-20_SampleA | [Gp0648941](https://gold.jgi.doe.gov/project?id=Gp0648941) | [Ga0558120](https://gold.jgi.doe.gov/analysis_project?id=Ga0558120) | [3300056831](https://img.jgi.doe.gov/cgi-bin/mer/main.cgi?section=TaxonDetail&page=taxonDetail&taxon_oid=3300056831) | [SAMN37864923](https://www.ncbi.nlm.nih.gov/sra/?term=SAMN37864923) | PacBio 6-10kb |
| AOia | 2021-01-27 | 638 | RS_2021-01-27_SampleA | [Gp0615591](https://gold.jgi.doe.gov/project?id=Gp0615591) | [Ga0558719](https://gold.jgi.doe.gov/analysis_project?id=Ga0558719) | [3300056759](https://img.jgi.doe.gov/cgi-bin/mer/main.cgi?section=TaxonDetail&page=taxonDetail&taxon_oid=3300056759) | [SAMN37864935](https://www.ncbi.nlm.nih.gov/sra/?term=SAMN37864935) | PacBio 6-10kb |
| UCTca | 2020-02-12 | 288 | RS_2020-02-12_SampleB | [Gp0648938](https://gold.jgi.doe.gov/project?id=Gp0648938) | [Ga0558117](https://gold.jgi.doe.gov/analysis_project?id=Ga0558117) | [3300056624](https://img.jgi.doe.gov/cgi-bin/mer/main.cgi?section=TaxonDetail&page=taxonDetail&taxon_oid=3300056624) | [SAMN37864947](https://www.ncbi.nlm.nih.gov/sra/?term=SAMN37864947) | PacBio 6-10kb |
| UCTca | 2020-07-02 | 429 | RS_2020-07-02_SampleB | [Gp0648940](https://gold.jgi.doe.gov/project?id=Gp0648940) | [Ga0558119](https://gold.jgi.doe.gov/analysis_project?id=Ga0558119) | [3300056825](https://img.jgi.doe.gov/cgi-bin/mer/main.cgi?section=TaxonDetail&page=taxonDetail&taxon_oid=3300056825) | [SAMN37864954](https://www.ncbi.nlm.nih.gov/sra/?term=SAMN37864954) | PacBio 6-10kb |
| UCTca | 2020-07-14 | 441 | RS_2020-07-14_SampleB | [Gp0615590](https://gold.jgi.doe.gov/project?id=Gp0615590) | [Ga0569290](https://gold.jgi.doe.gov/analysis_project?id=Ga0569290) | [3300056854](https://img.jgi.doe.gov/cgi-bin/mer/main.cgi?section=TaxonDetail&page=taxonDetail&taxon_oid=3300056854) | [SAMN37864920](https://www.ncbi.nlm.nih.gov/sra/?term=SAMN37864920) | PacBio 3kb |
| UCTca | 2021-01-20 | 631 | RS_2021-01-20_SampleB | [Gp0648942](https://gold.jgi.doe.gov/project?id=Gp0648942) | [Ga0558121](https://gold.jgi.doe.gov/analysis_project?id=Ga0558121) | [3300056625](https://img.jgi.doe.gov/cgi-bin/mer/main.cgi?section=TaxonDetail&page=taxonDetail&taxon_oid=3300056625) | [SAMN37864945](https://www.ncbi.nlm.nih.gov/sra/?term=SAMN37864945) | PacBio 6-10kb |
| UCTca | 2021-01-27 | 638 | RS_2021-01-27_SampleB | [Gp0615592](https://gold.jgi.doe.gov/project?id=Gp0615592) | [Ga0569289](https://gold.jgi.doe.gov/analysis_project?id=Ga0569289) | [3300056872](https://img.jgi.doe.gov/cgi-bin/mer/main.cgi?section=TaxonDetail&page=taxonDetail&taxon_oid=3300056872) | [SAMN37864943](https://www.ncbi.nlm.nih.gov/sra/?term=SAMN37864943) | PacBio 3kb |
| AO-G | 2021-06-24 | 35 | RS_2021-06-24_SampleA | [Gp0615593](https://gold.jgi.doe.gov/project?id=Gp0615593) | [Ga0569288](https://gold.jgi.doe.gov/analysis_project?id=Ga0569288) | [3300056853](https://img.jgi.doe.gov/cgi-bin/mer/main.cgi?section=TaxonDetail&page=taxonDetail&taxon_oid=3300056853) | [SAMN37864950](https://www.ncbi.nlm.nih.gov/sra/?term=SAMN37864950) | PacBio 3kb |
| AO-G | 2021-07-01 | 39 | RS_2021-07-01_SampleA | [Gp0648943](https://gold.jgi.doe.gov/project?id=Gp0648943) | [Ga0558122](https://gold.jgi.doe.gov/analysis_project?id=Ga0558122) | [3300056817](https://img.jgi.doe.gov/cgi-bin/mer/main.cgi?section=TaxonDetail&page=taxonDetail&taxon_oid=3300056817) | [SAMN37864928](https://www.ncbi.nlm.nih.gov/sra/?term=SAMN37864928) | PacBio 6-10kb |
| AO-G | 2021-08-19 | 88 | RS_2021-08-19_SampleA | [Gp0648945](https://gold.jgi.doe.gov/project?id=Gp0648945) | [Ga0558124](https://gold.jgi.doe.gov/analysis_project?id=Ga0558124) | [3300056599](https://img.jgi.doe.gov/cgi-bin/mer/main.cgi?section=TaxonDetail&page=taxonDetail&taxon_oid=3300056599) | [SAMN37864927](https://www.ncbi.nlm.nih.gov/sra/?term=SAMN37864927) | PacBio 6-10kb |
| AO-G | 2021-08-26 | 95 | RS_2021-08-26_SampleA | [Gp0615595](https://gold.jgi.doe.gov/project?id=Gp0615595) | [Ga0558717](https://gold.jgi.doe.gov/analysis_project?id=Ga0558717) | [3300056788](https://img.jgi.doe.gov/cgi-bin/mer/main.cgi?section=TaxonDetail&page=taxonDetail&taxon_oid=3300056788) | [SAMN37864951](https://www.ncbi.nlm.nih.gov/sra/?term=SAMN37864951) | PacBio 6-10kb |
| AO-G | 2021-10-20 | 150 | RS_2021-10-20_SampleA | [Gp0648947](https://gold.jgi.doe.gov/project?id=Gp0648947) | [Ga0558126](https://gold.jgi.doe.gov/analysis_project?id=Ga0558126) | [3300056626](https://img.jgi.doe.gov/cgi-bin/mer/main.cgi?section=TaxonDetail&page=taxonDetail&taxon_oid=3300056626) | [SAMN37864949](https://www.ncbi.nlm.nih.gov/sra/?term=SAMN37864949) | PacBio 6-10kb |
| AO-FF | 2021-0624 | 32 | RS_2021-06-24_SampleB | [Gp0615594](https://gold.jgi.doe.gov/project?id=Gp0615594) | [Ga0558718](https://gold.jgi.doe.gov/analysis_project?id=Ga0558718) | [3300056850](https://img.jgi.doe.gov/cgi-bin/mer/main.cgi?section=TaxonDetail&page=taxonDetail&taxon_oid=3300056850) | [SAMN37864937](https://www.ncbi.nlm.nih.gov/sra/?term=SAMN37864937) | PacBio 6-10kb |
| AO-FF | 2021-07-01 | 42 | RS_2021-07-01_SampleB | [Gp0648944](https://gold.jgi.doe.gov/project?id=Gp0648944) | [Ga0558123](https://gold.jgi.doe.gov/analysis_project?id=Ga0558123) | [3300058930](https://img.jgi.doe.gov/cgi-bin/mer/main.cgi?section=TaxonDetail&page=taxonDetail&taxon_oid=3300058930) | [SAMN37864948](https://www.ncbi.nlm.nih.gov/sra/?term=SAMN37864948) | PacBio 3kb |
| AO-FF | 2021-08-19 | 91 | RS_2021-08-19_SampleB | [Gp0648946](https://gold.jgi.doe.gov/analysis_project?id=Ga0558716) | [Ga0558125](https://gold.jgi.doe.gov/analysis_project?id=Ga0558125) | [3300056927](https://img.jgi.doe.gov/cgi-bin/mer/main.cgi?section=TaxonDetail&page=taxonDetail&taxon_oid=3300056927) | [SAMN37864933](https://www.ncbi.nlm.nih.gov/sra/?term=SAMN37864933) | PacBio 3kb |
| AO-FF | 2021-10-05 | 135 | RS_2021-10-05_SampleB | [Gp0615599](https://gold.jgi.doe.gov/project?id=Gp0615599) | [Ga0558716](https://gold.jgi.doe.gov/analysis_project?id=Ga0558716) | [3300056999](https://img.jgi.doe.gov/cgi-bin/mer/main.cgi?section=TaxonDetail&page=taxonDetail&taxon_oid=3300056999) | [SAMN37864946](https://www.ncbi.nlm.nih.gov/sra/?term=SAMN37864946) | PacBio 6-10kb |

**Table S2. Protologue Table for *Candidatus* Accumulibacter jenkinsii**

| Species name | *Candidatus* Accumulibacter jenkinsii |
| --- | --- |
| Genus name | *Candidatus* Accumulibacter |
| Specific epithet | jenkinsii |
| Type species of the genus | *Candidatus* Accumulibacter phosphatis (UW1) |
| Genus status | Candidatus |
| Species etymology | jen.kin’si.i. N.L. gen. n., named after Dr. David Jenkins, recognizing his many contributions to our understanding of activated sludge microbiology. |
| Species status | sp. nov. |
| Designation of the type MAG | UW20 |
| MAG/SAG accession number | IMG ID: [3300056999_154](https://img.jgi.doe.gov/cgi-bin/mer/main.cgi?section=MetagenomeBinDetail&page=bindetail&bin_oid=3300056999_154) |
| Genome status | High-quality draft |
| Genome size | 4,517,007 |
| GC mol % | 63.6 |
| Country of origin | USA |
| Region of origin | Wisconsin |
| Source of sample | Pilot-Scale Bioreactor |
| Sampling date  Geographical location | October 5, 2021  Madison Metropolitan Sewerage District |
| Latitude | 43.0355 N |
| Longitude | 89.3566 W |
| Depth | - |
| Altitude | - |
| Temperature of the sample | - |
| pH of the sample | - |
| Relationship to oxygen | Facultative anaerobe |
| Energy metabolism | Polyphosphate-accumulating organism |
| Assembly (no. of samples) | 1 sample |
| Sequencing technology | PacBio |
| Binning software used | metaBAT2, version 2.15 |
| Assembly software used | metaFlye, version 2.8.1 |
| Habitat | Biological nutrient removal bioreactor |
| Miscellaneous, extraordinary features relevant for the description | Present in biological nutrient removal wastewater treatment plant processes achieving efficient enhanced biological phosphorus removal (EBPR) when operated with low dissolved oxygen concentrations |

**REFERENCES**

1. Jain C, Rodriguez-R LM, Phillippy AM, Konstantinidis KT, Aluru S. 2018. High throughput ANI analysis of 90K prokaryotic genomes reveals clear species boundaries. Nature communications 9:5114.

2. Petriglieri F, Singleton CM, Kondrotaite Z, Dueholm MKD, McDaniel EA, McMahon KD, Nielsen PH, McGrath J. 2022. Reevaluation of the Phylogenetic Diversity and Global Distribution of the Genus mSystems 0:e00016-22.

3. McDaniel EA, Moya-Flores F, Beach NK, Camejo PY, Oyserman BO, Kizaric M, Khor EH, Noguera DR, McMahon KD, Hug LA. 2021. Metabolic Differentiation of Co-occurring Accumulibacter Clades Revealed through Genome-Resolved Metatranscriptomics. mSystems 6:e00474-21.
